# 4D Printing Applications in the Development of Smart Cardiovascular Implants

**DOI:** 10.3389/fbioe.2022.873453

**Published:** 2022-05-25

**Authors:** Fatemeh Kabirian, Petra Mela, Ruth Heying

**Affiliations:** ^1^ Cardiovascular Developmental Biology, Department of Cardiovascular Sciences, KU Leuven, Leuven, Belgium; ^2^ Medical Materials and Implants, Department of Mechanical Engineering and Munich School of BioEngineering, Technical University of Munich, Munich, Germany

**Keywords:** 4D printing, 3D printing, bioprinting, cardiovascular, cardiac patches, vascular grafts, vascular stents, biomaterials

## Abstract

Smart materials are able to react to different stimuli and adapt their shape to the environment. Although the development of 3D printing technology increased the reproducibility and accuracy of scaffold fabrication, 3D printed scaffolds can still be further improved to resemble the native anatomy. 4D printing is an innovative fabrication approach combining 3D printing and smart materials, also known as stimuli-responsive materials. Especially for cardiovascular implants, 4D printing can promisingly create programmable, adaptable prostheses, which facilitates implantation and/or create the topology of the target tissue post implantation. In this review, the principles of 4D printing with a focus on the applied stimuli are explained and the underlying 3D printing technologies are presented. Then, according to the type of stimulus, recent applications of 4D printing in constructing smart cardiovascular implants and future perspectives are discussed.

## 1 Introduction

Cardiovascular structures such as cardiac valves and vascular branches have a complicated architecture potentially requiring a customized design and fabrication of implants. Recent research showed the potential of 3D printing for the development of cardiovascular implants that can potentially be fabricated in a personalized manner according to the patient’s anatomy (Farzin et al., 2018; Kabirian et al., 2019; Pedrotty et al., 2019; Kabirian et al., 2020; Thomas et al., 2020; Asulin et al., 2021).

Despite promising progress in the application of 3D printing and bioprinting in the fabrication of medical devices, conformational changes of the printed structure according to the individual patient’s anatomy after implantation are of interest, especially for cardiovascular prostheses (Ashammakhi et al., 2018). 4D printing is a further step in the evolution of the 3D printing approach in which the printed structure changes its shape, function, and/or properties over time (Quanjin et al., 2020).

**FIGURE 1:**
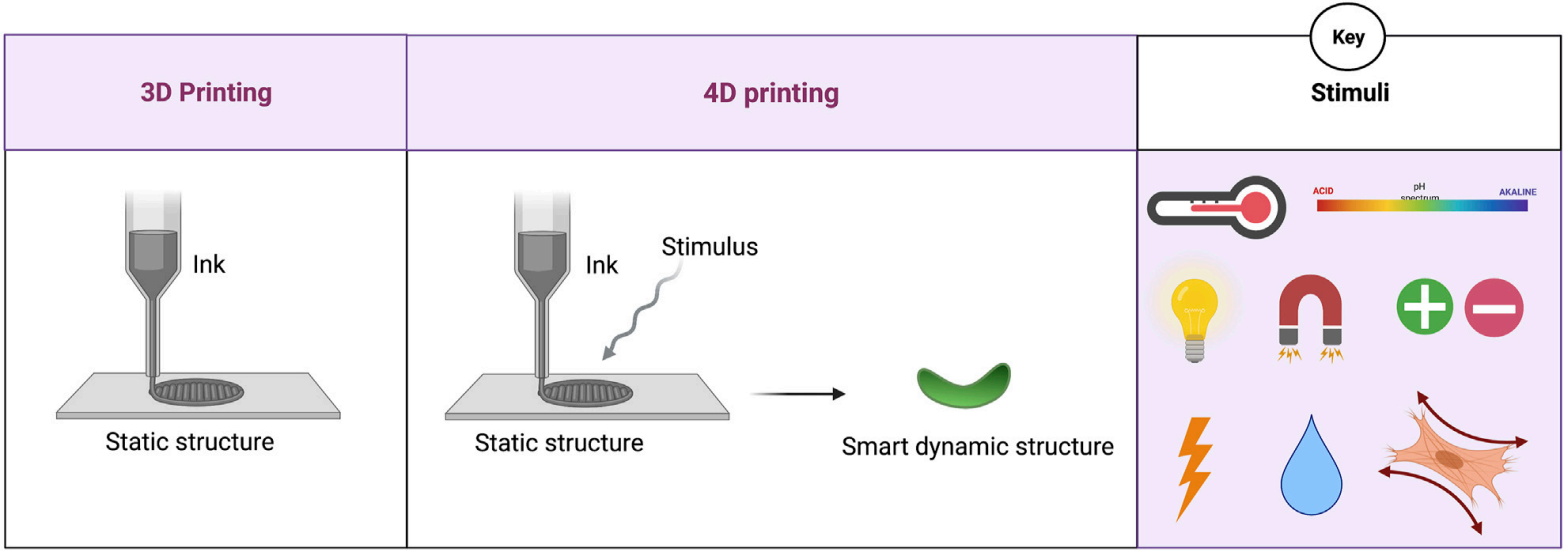
Schematic illustration of 3D printing and 4D printing approaches. Physical, chemical, and biological stimuli are summarized in the key box including temperature, light, electrical and magnetic field, moisture, pH, ions, and cell traction.

4D printing is defined by using smart materials, also known as stimuli-responsive materials, to fabricate implants by applying 3D printing and bioprinting technologies (Figure 1) (Simińska-Stanny et al., 2022). Therefore, the 4D printed device is similar to the 3D printed one while the fourth dimension, the time, allows the smart material to become dynamic and to transform upon a stimulus. Smart materials are classified into shape memory materials (SMMs) and shape changing materials (SCMs). SMMs recover their original shape in response to the stimulus. In contrast, SCMs respond to stimuli by showing a temporary shape and return to their original shape after stimuli removal (Patil and Song, 2017). For example, shape memory polymers (SMPs), commonly used in 4D printing, have the ability to revert back to the original shape in response to stimuli by changing from a rigid polymer to an elastic state and coming back again to the rigid state. Between these states, a large reversible change of elastic modulus is observable (Pei and Loh, 2018). Therefore, 4D printed materials can work to reversibly respond to environmental stimuli (Agarwal et al., 2021a; Wang et al., 2021a). Currently, 4D printed cardiovascular implants are tested *in vitro* and in animal models. This article aims to overview the recent developments in the use of 4D printing in the cardiovascular field by classifying the applications based on the type of stimulation used. The novel aspect of our work is the focus on recapitulating the applications of 4D printing in the fabrication of cardiovascular implants. We aimed to categorize the 4D printed cardiovascular implants based on the applied stimuli to highlight the current application of each stimulus and the general future perspective.

## 2 3D Printing and Bioprinting Technologies

**FIGURE 2:**
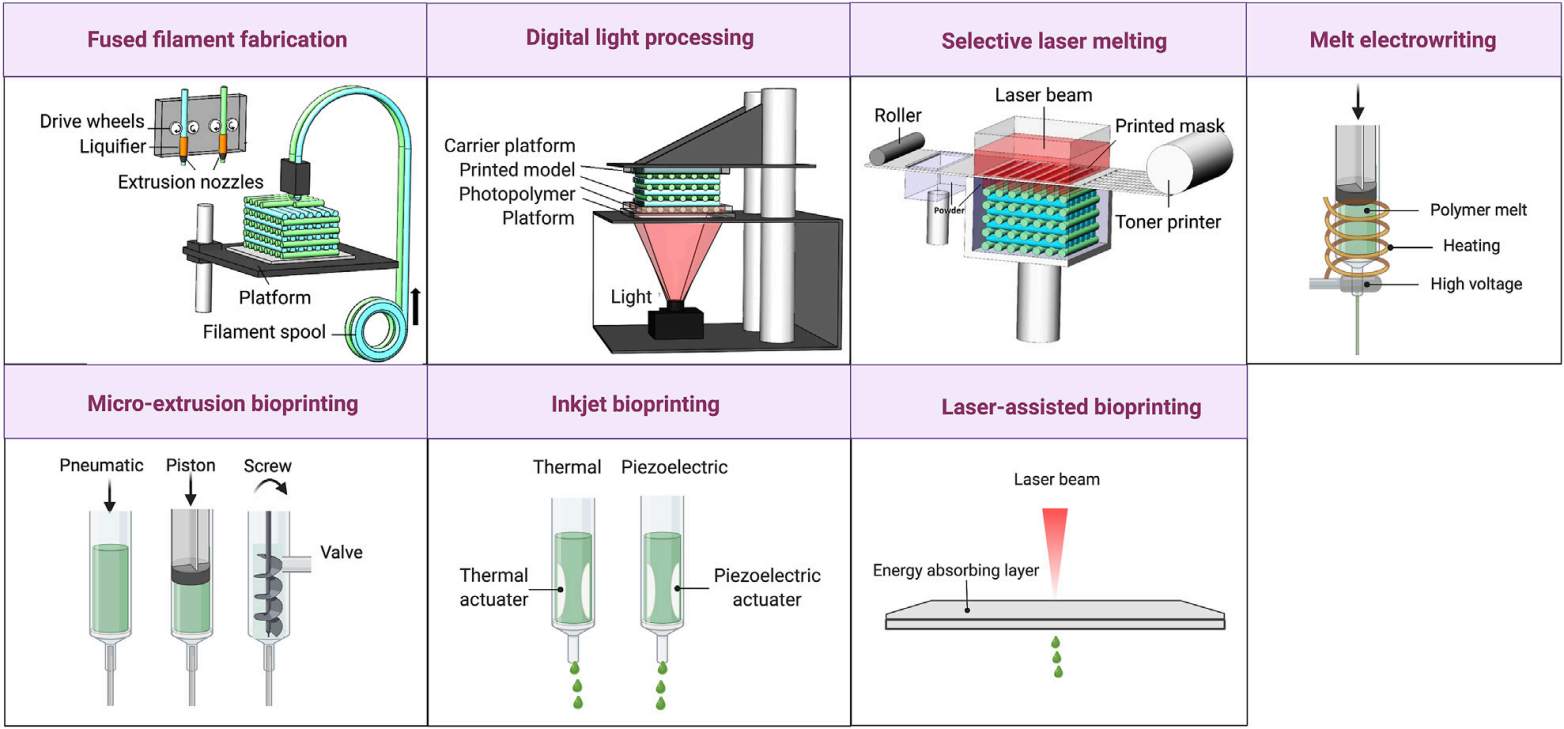
The schematic representation of 3D printing and bioprinting techniques. 3D printing techniques such as fused filament fabrication (FFF), digital light processing (DLP), selective laser melting (SLM), and melt electrowriting (MEW). FFF is an extrusion approach for melted thermoplastic polymers. DLP is applicable for photopolymers. In SLM which is applicable for powder materials, a laser beam fuses the particles. MEW is a nozzle-based method to extrude solvent-free polymer fibers in a high-voltage field. Bioprinting methods are including micro-extrusion, inkjet, and laser-assisted bioprinting. Micro-extrusion works based on pneumatic or mechanical (piston or screw) dispension. Inkjet printing ejects droplets of material by thermal or piezoelectric pressure. In laser-assisted printing, the material absorbs the laser energy and is ejected as a jet of bioink. Reproduced with permission of Touri et al. (2019).

3D printing, also known as additive manufacturing, is a layer-by-layer manufacturing approach according to the digital 3D model. Due to the high reproducibility, accuracy, and cost effectiveness, it has various applications in the fabrication of medical devices. As represented in Figure 2, fused filament fabrication (FFF), digital light processing (DLP), and selective laser melting (SLM) are important methods of 3D printing used for manufacturing medical devices. FFF works by extrusion of melted thermoplastic filaments through a nozzle. The resulting objects are solvent free with a resolution determined by the nozzle diameter (e.g., 100 µm) (Wallin et al., 2018). DLP uses a projection of ultraviolet or visible light to crosslink photocurable resins in a vat. While DLP is capable to fabricate microscale components with a resolution of 1 μm, the application is limited to photo-sensitive polymers. DLP is a more rapid method with a higher resolution compared to extrusion printing which can be used to fabricate precise structures of vessels such as bifurcations. For example, the DLP (Lumen X) printer showed a 74% success rate to fabricate bifurcations while the success rate was 60% for the extrusion approach (Tomov et al., 2021). Quantum X bio is another recently developed light-based printing method which is appropriate for cardiovascular applications.

SLM is a powder-based additive manufacturing method in which powder particles are locally fused via thermal energy introduced by a laser beam. The resolution (15 µm) is mainly related to the powder particle size and the laser beam diameter (Nagarajan et al., 2019). Although SLM is applicable for the production of complex morphologies with a porous structure, limited raw materials are available in powder form (Touri et al., 2019). Melt electrowriting (MEW) is a combination of melt-electrospinning and additive manufacturing to enable the controlled deposition of polymer fibers according to predefined patterns from an extruded solvent-free jet assisted by a high-voltage electric field. MEW is a high-resolution method capable to produce fibers with diameters ranging from nanometers to tens of micrometers (Saidy et al., 2020).

By incorporating cells into the 3D printing approach, 3D bioprinting has been developed. Bioprinting with bioinks, illustrated in Figure 2, has been used in three main methods: micro-extrusion, inkjet-assisted, and laser-assisted bioprinting. Micro-extrusion bioprinting is the most common approach that works based on pneumatic piston-driven or screw-driven dispensing. Although the micro-extrusion is a simple and reproducible method that allows printing highly cellular bioinks and various biomaterials, the printing speed and resolution are low (Kabirian and Mozafari, 2020). Moreover, since high viscosity (10–10^6^ Pa_*_s) is required to maintain the morphology after extrusion, the high shear forces result in lower cellular viability (75–90%) than obtained with inkjet-assisted or laser-assisted bioprinting (> 90%) (Bedell et al., 2020). Bioinks with shear-thinning properties are a solution to this issue. Inkjet bioprinting generates a non-continuous flow of bioink by ejecting droplets through bubble producing or vibration of a piezoelectric actuator (Li et al., 2020). Thermal inkjetting with 4–10°C enhancement of temperature during 2 μs is more cytocompatible since the piezoelectric method can lead to damage of the cell membrane and to cell lysis (Murphy and Atala, 2014). While the inkjet approach offers a fast speed, it requires low viscosities (< 10 mPa_*_s) and low cellular densities. The bioink needs to be crosslinked after deposition and the shear stress should be kept to less than 10 kPa to keep cells viable (Bedell et al., 2020). Laser-assisted bioprinting is based on the laser-induced forward transfer principles (Derakhshanfar et al., 2018). In this type of bioprinters, a pulsed laser is absorbed by an energy-absorbing layer to elicit a phase change leading to the ejection of a jet of bioink. The resulting cellular viability is higher in this method than in inkjet printing since no nozzle is used and no shear stress is applied. The resolution is also higher in this method due to the use of picoliter-sized droplets and the use of the laser (Keriquel et al., 2017). Although laser-assisted bioprinting is able to print both liquid and solid bioinks and offers high resolution, it is an expensive procedure due to the need for a high precision laser and the laser irradiation may cause thermal damage. The viscosity of bioink in this method should be in the order of 100 mPa_*_s and secondary crosslinking is required (Bedell et al., 2020).

## 3 4D Printing of Cardiovascular Implants

In the future, 4D printed devices have a great potential for improved functionality compared to the commercially available implants used in the treatment of various cardiovascular diseases. Current efforts are made in the treatment of myocardial infarction (MI), a common cardiac disease in which the cardiac muscle is limited to repair and regenerate itself. Therefore, cardiac patches are developed to support the mechanical function of the heart. In a recently developed approach, cardiac patches have been shown to actively initiate tissue regeneration and remodeling for which they need to keep their alignment with the individual heart curvature. The 4D printing enables the production of patches out of smart materials adjustable to the specific cardiac architecture (Miao et al., 2018; Cui et al., 2020). Moreover, with the development of foldable, origami-based smart cardiovascular implants, it is possible to place the patches with minimal invasive access via two small incisions (Mei et al., 2021). Overall, in MI, 4D printing may allow a completely new strategy of treatment. Another potential for 4D printing is seen in the Interventional closure of the left atrial appendage (LAA) in patients with atrial fibrillation (AF) which is now frequently done in the elderly patient to prevent them from their high risk of stroke (15–20%). Considering the side effects of anticoagulant drugs, devices are constructed to fill the lumen of the LAA to prevent thrombus formation. 4D printing allows the production of customized devices to adapt to the individual shape of the LAA of each patient (Lin et al., 2021).

The shape or function of a printed device from smart materials can change by an external stimulus (Willemen, Morsink, Veerman, da Silva, Cardoso, Souto, Severino; Wu et al., 2021) over time improving the implants functionality (Li et al., 2020). Also, the invasiveness of for example, surgery can be reduced by using 4D printed devices by creating a device which only expands to its full size after implantation (Zhang et al., 2021). Various materials and physical, chemical, and biological stimuli have been employed in 4D printing.

### 3.1 Physical Stimuli

Smart materials change their shape and properties in response to physical stimuli such as temperature, light, electric, and magnetic fields transforming the shape of stimuli-responsive materials.

#### 3.1.1 Temperature

**FIGURE 3:**
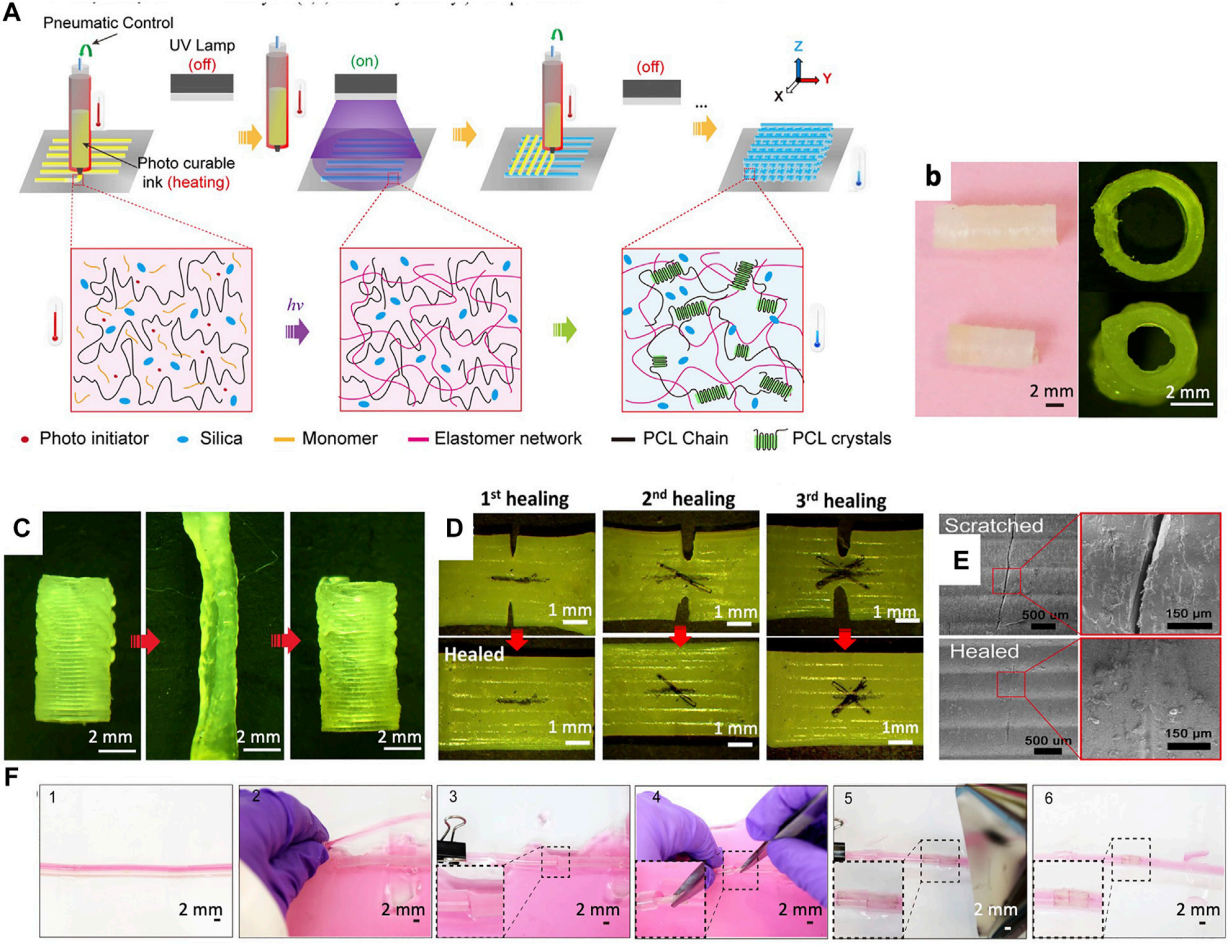
Fabrication steps and investigations of 4D printed vascular grafts. **(A)** Schematic representation of the 3D printing of vascular grafts from UV curable composite ink composed of crystalline linear chain and crosslinked network and the structural changes during this process. **(B)** Photograph (left) and microscopic image (right) of two different inner/outer diameters of printed vascular grafts with the same length of 10 mm. **(C)** Stretching and compressing of vascular conduits by thermal stimulation at 70°C (above PCL T_m_) to the temporary shape with half of the initial diameter and shape recovery after cooling down (below PCL T_m_). **(D)** Three healing cycles of the printed structures by cooling down in the air after heating at 80°C for 20 min **(E)** Scanning electron microscopy (SEM) observation of the scratched and healed part of the sample. **(F)** (1) The 4D printed blood vessel (2) was cut to be prepared for implantation. (3) By clamping the bleeding was stopped. (4) Implantation of the 4D printed graft in the crack region. (5) Shape recovery and attachment of the 4D printed graft to the vasculature by heating. (6) Blood circulation after vascular connection. Reproduced with permission of Kuang et al. (2018).

Temperature is one of the most commonly used stimuli in 4D printing. Thermo-responsive shape memory implants are designed to transform their shape to their final morphology after implantation at body temperature. Initially, according to the target geometry, the temperature of the scaffold is elevated above the transition temperature (T_trans_) for shape programming of the implant. T_trans_ in semi-crystalline polymers and amorphous polymers is equal to the melting temperature (Tm) or glass transition temperature (Tg), respectively. At T_trans_, the scaffold deforms to a temporary shape appropriate for implantation. This temporary shape is fixed by cooling down to a temperature below T_trans_ which will recover to the permanent shape after implantation by heating above T_trans_ (Kuang et al., 2019). Therefore, to avoid causing frostbite or scald to the tissues, the challenge is to keep T_trans_ between 20 and 37°C as this is required for the shape programming temperature range. In a recent study, it has been indicated that synthesized poly(glycerol dodecanoate) acrylate (PGDA) is an appropriate material that can be 4D printed with T_trans_ 20–37°C. Shape memory vascular stents with mechanical and geometrical adaptability were printed out of PGDA and then photo-crosslinked. Next, they were programmed to a temporary compact shape for delivery above T_trans,_ and then after deployment by heating above the T_trans_, the stored energy was released and the stent recovered the target shape. Interestingly, with the same approach, 4D printed vascular grafts were developed and implanted into a mouse aorta. By ligating the two ends of the vessel, the blood of the target vessel was drained to cool down the section and keep the temperature lower than the body temperature. After implantation and by allowing blood flow inside the graft, the shape of the graft was recovered to the final and permanent morphology. The 4D printed vascular stents and grafts exhibited a high recovery ratio of 98% at 37°C, cycling stability, and rapid recovery time which can open new horizons for the next generation of vascular implants. This is a great advantage compared to the commercially available vascular stents and grafts which have fixed dimensions and mechanical properties (Zhang et al., 2021). 4D printed, curved films (aligned to the heart curvature) with a thickness smaller than 300 µm were also fabricated from soybean oil epoxidized acrylate (SOEA) by a photolithographic-stereolithographic-tandem strategy (PSTS) printing technique. These shape memory films seem appropriate for the application in cardiac tissue engineering increasing cardiomyogenic differentiation of stem cells. The aim of these 4D printed films is to optimize the integration within damaged heart tissue and to minimize the invasiveness of the surgical delivery and subsequent trauma by *in situ* shape change which can potentially improve patient comfort in the future. The thin films were stimulated to bend or roll by being warmed up at body temperature. In this study, UV crosslinking resulted in generating the internal strength during a photolithography process. The UV exposure attenuated through the thickness of gel production resulting in a crosslink density gradient. Differential swelling and a bending moment occur which initiates self-folding (Miao et al., 2018). In another approach, 4D printed vascular grafts were fabricated from a composite ink prepared by photho-curable resin [aliphatic urethane diacrylate (AUD) and n-butyl acrylate (BA)], semi-crystalline polycaprolactone (PCL) and nanoparticles such as fumed silica (Figure 3). The rheology of the composite ink was tuned by nanoparticles to achieve printability and self-healing (structural restoration and functional recovery) and shape memory behaviors after direct-ink-write (DIW) printing. Due to the self-healing behavior, the 4D printed vascular grafts could heal microcracks and even notched gaps by the entanglement of PCL chains (restoration of the mechanical properties) between urethane chains. By thermal stimulation, shape memory behavior can also help to close large cracks (Figures 3–E). These self-healing properties are especially important after implantation of vascular grafts in which the graft can be damaged or perforated during the operation. In this case, self-healing can prevent constant bleeding and further surgical repair. Moreover, the shape memory-self-healing vascular grafts can eliminate the need for surgical suturing which is a time-consuming approach. In this study, the *in vitro* monitoring was performed with red colored water (Kuang et al., 2018). In another approach, biodegradable 4D printed vascular stents were fabricated from a photocrosslinkable ink from a star polymer composed of a β-cyclodextrin (βCD) core and 21 PCL arms with an acrylate end group. Interestingly, the 4D printed stents demonstrated appropriate mechanical functions, such as tensile strength, elasticity, and burst pressure comparable to the human great saphenous vein. The stents were also designed to deliver sustained paclitaxel release. The aim of these stents was to be implanted small in size (diameter of 5 mm) to reduce the surgical damage and recover the target shape after deployment upon a thermal stimulus (Zhou et al., 2021).

#### 3.1.2 Light

Light can change the structure of photoresponsive materials by various mechanisms. Common processes are photocrosslinking (photoinduced crosslinking) and photodegradation (or photocleavage) which is a temporal or spatial reduction of the crosslinking by light exposure (Kloxin et al., 2009; Guvendiren and Burdick, 2012). Also, light-induced stereoisomerism, a form of isomerization induced by photoexcitation (Van Oosten et al., 2009), and light-induced hydrophobicity (change of wettability in response to light) (Ter Schiphorst et al., 2016) are of importance. In addition, photothermal effects characterized by photoexcitation of the material resulting in the production of thermal energy can change the structure of materials (Wang et al., 2020).

**FIGURE 4:**
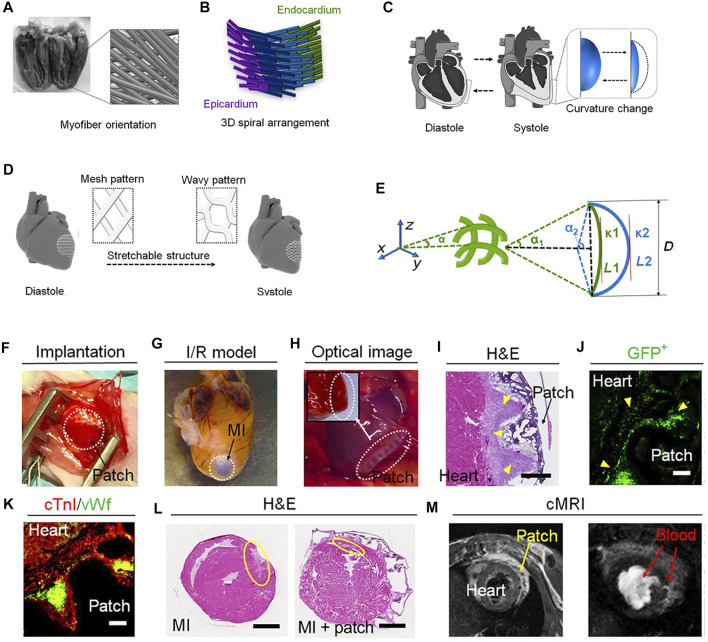
Design, fabrication, and *in vivo* studies of 4D cardiac patches. **(A)** Myofiber orientation of the left ventricular wall from +60° to −60° which rotates **(B)** left-handed from epicardium to the right-handed in the endocardium. **(C)** Cardiac curvatures in diastole and systole. **(D)** CAD design of the 3D heart architecture during stretching. **(E)** Geometric model of fibers in printed patches in which α represents the angle and L the length of the fiber, D the special displacement, and κ 1 and 2 the ventricular curvature in systole and diastole, respectively. **(F)** Implanted 4D printed patch and **(G)** MI heart model after 4 months of implantation. **(H)** Firm attachment of an implanted cellularized patch after 3 weeks **(I)** H&E staining of the cellularized patch after 3 weeks of implantation indicating a high concentration of cells (yellow arrows and scale bar: 400 µm). **(J)** Fluorescent image of GFP + hiPSC-CMs after 3 weeks of implantation demonstrating high viability and engraftment (scale bar: 100 µm). **(K)** Immunofluorescence staining of cTnIand vWf verifying the presence of hiPSC-CMs and hECs on the cellularized patches after 3 weeks of implantation (scale bar: 100 µm). **(L)** Comparison of infarct size indicated by yellow circles, mouse model control (left, ∼ 8.4 ± 1.1%) and with patch implantation (right, ∼ 3.8 ± 0.7%) after 10 weeks. **(M)** MRI imaging of the heart with implanted patch after 10 weeks. Reproduced from Cui et al. (2020).

Light which is controllable by specific pattern and photo exposure energy can interact with photoresponsive polymers (Kuang et al., 2019). Photoresponsive materials convert light energy into mechanical forces in response to a wide range of wavelengths (Wei M. et al., 2017). Near-infrared (NIR) light-sensitive 4D printed cardiac patches have been developed to fabricate scaffolds with a reprogramming capacity. The aim was to adjust them to the curvatures of the heart after myocardial infarction (MI) making them an interesting product for commercialization with appropriate personalized capacities. DLP printing was used to print a photo crosslinkable NIR light-sensitive ink material, composed of polyethylene glycol diacrylate (PEGDA) and graphene nanoplatelets. The 4D printed cardiac patches revealed the capacity for spatiotemporal transformation by photothermal stimulation. In the photothermal process, the NIR light remotely controls the shape changing of the nanocomposite structure in which graphene nanoparticles absorb the heat from NIR and raise the temperature (Wang et al., 2021b). The 4D cardiac patches were also printed from GelMA and a PEGDA-based photocurable bioink by beam-scanning stereolithography (SL) printing technique (Figure 4) and implanted in a mouse model. The aim was to achieve the same heart’s curvature by self-morphing of these patches. In addition, the patches were designed to switch between the wavy and mesh pattern based on the systolic and diastolic cycles (Figures 4–D). This switching was induced by a light-induced internal stress (affecting the physicochemical properties of the hydrogel by photo crosslinking) resulting in a solvent-induced material relaxation (reversible shape transformation by solvent swelling). Human induced pluripotent stem cell–derived cardiomyocytes (hiPSC-CMs) indicated a high engraftment onto the patches after 3 weeks (Figures 4–J). *In vivo* results revealed a firm attachment of the patches to the epicardium and vascular infiltration obtained after 3 weeks of implantation in a chronic MI model in mice (Figure 4) (Cui et al., 2020).

#### 3.1.3 Electric and Magnetic Fields

Electric and magnetic fields alter the structure of a material by their initiated thermal effects. Materials sensitive to the electric field are usually polyelectrolyte hydrogels that react by swelling, shrinking, erosion, or bending upon the electric stimulus. Doping hydrogels with conductive polymers such as polypyrrole and polythiophene is another approach to obtain hydrogels responsive to the electric field. Carbon-based nanoparticles such as carbon nanotubes and graphenes are alternative options to make materials electro-sensitive (Ashammakhi et al., 2018).

Magnetic field responsive materials are containing ferromagnetic or paramagnetic micro or nanoparticles. Due to the high surface-to-volume ratio, nanoparticles display different properties than bulk materials (Patade et al., 2020). Self-heating is one of the properties that magnetic nanoparticles exhibit based on their particle size. This property is due to loss of hysteresis and magnetic relaxation (Kobayashi et al., 2010; Nakamura et al., 2012). According to the Néel-Brown model, the applied magnetic energy is converted into heat, also called self-heating, which allows the adaptation of the used material (Wang et al., 2021a). This approach has been used for the construction of biodegradable patient-specific left atrial appendage occluders (LAAO). The LAAOs were 4D printed from PLA and a Fe_3_O_4_ magnetic nanocomposite. The usage of magnetic nanoparticles enabled the self-heating remote controllable 4D transformation. The 4D printed LAAOs were programmed to exhibit a straight shape with a small cross-section area to temporarily facilitate the implantation. Two types of LAAOs, single-layer and double-layer, were developed based on a bio-inspired hex88 network pattern with the closest stress-strain response to LAA. Interestingly, the transformation time of double-layered LAAOs was shorter than single-layered LAAOs. This might be due to the higher concentration of magnetic nanoparticles and the lower degree of freedom in the double-layered LAAOs. In this study, the magnetism-induced recovery in a magnetic field took a longer time compared with heat-induced transformation in a hot water bath. This can be explained by the fact that nanoparticles as self-heaters can only transfer the heat within the LAAO structure, while in heat-inducing transformation the whole LAAO was immersed in the liquid and therefore the heat transfer was faster. The most important advantage of these smart LAAOs, compare to commercial nitinol-based occlusion implants, is the biodegradability which together with the potential for being customized and adapted to the patient’s tissue deformation minimizes undesirable complications such as corrosion and allergy (Lin et al., 2021). Self-expandable biodegradable vascular stents were also 4D printed from a UV-crosslinkable ink composed of PLA and Fe_3_O_4_ magnetic nanoparticles. These personalized stents were fabricated by DIW printing at 80°C (above Tg) and are able to re-expand the blood vessels with minimum invasiveness (Wei H. et al., 2017).

### 3.2 Chemical Stimuli

Chemical stimuli include the moisture, pH, and redox state of metal ions. Humidity works by swelling, temperature induces changes in crystallinity and hydrophobicity. pH alteration induces variations in electrostatic interactions and the redox state of metal ions controls the oxidative state of the metal ions.

#### 3.2.1 Humidity

Transformation of moisture responsive materials can be stimulated by water. Poly(ethylene glycol) (PEG) and hydrogels are the most common moisture responsive materials suitable for 4D printing. For example, a humidity responsive and thermo-responsive nanocomposite was developed from PCL, PEG, and cellulose nanocrystals (CNCs) nanofillers. Immersion of this nanocomposite strip inside water at 37°C caused shape recovery by swelling and water absorption (Liu et al., 2015). It is necessary to control the swelling of these materials in the transition process to keep the optimal integrity of the 4D printed scaffold (Amukarimi and Mozafari, 2021).

#### 3.2.2 pH

Since pH is adjustable and considered a significant parameter in different parts of the human environment, pH responsive polymers and hydrogels are applicable for 4D printing. The pH responsive polymers are classified into two groups, the ones with acidic or basic groups (Amukarimi and Mozafari, 2021). PH sensitive materials adapt to pH variations by swelling and de-swelling based on the chemical groups of the polymer such as amine and carboxylic acid, which release or absorb protons (Morouço et al., 2020).

#### 3.2.3 Ions

Ions are the common stimulus to change the swelling ratio of hydrogels. Hydrogel networks can directly interact with ions or are affected by osmotic pressure gradients due to ion concentration imbalance (Imrie and Jin, 2022). Biodegradable hollow tubes applicable as vascular grafts were 4D printed from methacrylated alginate and hyaluronic acid hydrogels. The reversible shape transformation was programmed by sensitivity to Ca^2+^ ions due to the presence of carboxylic groups in the chains of the polymeric matrix. By this approach, it was possible to create blood vessels with a small diameter of 20 µm which is unique to this technique. Another capacity of this fabrication approach is its ability to keep the shear forces in the lower range for the fabrication of small diameters (Kirillova et al., 2017).

### 3.3 Biological Stimuli

Biological stimuli such as enzymes, biomolecules, and cell traction forces are the last group of stimuli in this review. Multiple enzymes are specific molecules regulating different biological responses such as protein expression. Therefore, enzyme-responsive materials are attractive for application in 4D printing. Enzymes can induce degradation of the biomaterials leading to the break down of the implant after fulfilling the function which is of advantage in tissue engineering applications with a temporary aim. Biomolecules such as glucose are also modulating biological responses. For example, glucose responsive materials can play an important role in glucose monitoring and insulin delivery in diabetes mellitus patients (Kanu et al., 2019; Lui et al., 2019). Cells are exposed to a dynamic environment with various contractile forces present *in vivo.* These play an important role in tissue healing and regeneration, known as mechanotransduction (Lui et al., 2019; Agarwal et al., 2021b).

The so-called cell origami technique is based on the cell traction force approach that can be applied in 4D printing by inducing shape transformation using contractile forces of cells. To communicate with the adjacent cells and to re-organize the extracellular matrix (ECM), the adhered cells on the surface generate forces by the intracellular actin polymerization and interaction of actin and myosin. In this approach, cells actively fold themselves from a 2D to a 3D structure (Serpooshan et al., 2018). For example, in the cell origami approach, endothelial cells adhere to the connected plates and make tubular structures by cell traction forces. Since the plates are connected by flexible joints, cell traction forces result in folding of the plates until blocking of microplates (Kuribayashi-Shigetomi et al., 2012; Gao et al., 2016). The application of the cell origami method in 4D printing is still not well developed and by involving multiple cell types, complex cell-laden 4D printed constructs can develop and mimic the native tissue (Amukarimi and Mozafari, 2021). Table 1.

**TABLE 1:** 4D printed cardiovascular implants classified according to the stimulus.

3D Printing strategy	Ink	Stimulus	Application	Ref
*Physical stimulus*				
FFF	PGDA	Temperature	Shape memory vascular graft	Zhang et al. (2021)
PSTS	SOEA	Temperature	Shape memory thin film for integration with the damaged heart tissue and minimizing the invasiveness of the operation	Miao et al. (2018)
DIW	AUD, BA, PCL, and fumed silica nanoparticles	Temperature	Self-healing and shape memory vascular grafts able to heal microcracks and notched gaps eliminating the need for surgical suturing	Kuang et al. (2018)
DIW	βCD, PCL, and paclitaxel	Temperature	Biodegradable vascular stents to be implanted in a compressed size and recover the target shape after deployment to reduce the surgical damage	Zhou et al. (2021)
DLP	PEGDA and graphene nanoplatelets	Light	Adjustable scaffolds with the curvatures of the heart after MI making them an appropriate personalized product for commercialization	Wang et al. (2021b)
Beam-scanning stereolithography	GelMA and PEGDA	Light	Cardiac patches to attach to the epicardium after MI	Cui et al. (2020)
FFF	PLA and Fe3O4 magnetic nanocomposite	Magnetic fields	Biodegradable patient-specific left atrial appendage occluders	Lin et al. (2021)
DIW	PLA and Fe3O4 magnetic nanocomposite	Magnetic fields	Personalized self-expandable biodegradable vascular stents	Wei H. et al. (2017)
FFF	Commercially available flexible thermoplastic copolyester elastomer	Temperature	Self-expandable biodegradable vascular stents	Cabrera et al. (2017)
FFF	PLA	Temperature	Self-expandable biodegradable vascular stents	Jia et al. (2018)
DIW	Poly(d,l-lactide-co-trimethylene carbonate)	Temperature	Self-expandable biodegradable vascular stents	Wan et al. (2019)
FFF	PLA	Temperature	Self-expandable biodegradable vascular stents	Wu et al. (2018)
*Chemical stimulus*				
FFF	Methacrylated alginate and hyaluronic acid	Calcium ions	Biodegradable vascular grafts with internal diameter of 20 µm	Kirillova et al. (2017)
*Biological stimulus*				
-	Parylene microplates and cells	Cell traction force	Self-folding cell-laden microstructures	Kuribayashi-Shigetomi et al. (2012)

FFF, fused filament fabrication; PGDA, poly(glycerol dodecanoate) acrylate; PSTS, photolithographic-stereolithographic-tandem strategy; SOEA, soybean oil epoxidized acrylate; AUD, aliphatic urethane diacrylate; BA, n-butyl acrylate; PCL, polycaprolactone; βCD, β-cyclodextrin; DLP, digital light processing; PEGDA, polyethylene glycol diacrylate; MI, myocardial infarction; GelMA, gelatin methacrylate; PLA, polylactic acid.

## 4 Concluding Remarks and Future Perspectives

4D printing of cardiovascular implants shows promising progress in current *in vitro* experiments and animal studies. Actual efforts indicate a potential benefit of this technology especially in the development of vascular grafts, stents, and devices to close the LAA. Also, cardiac patches to support tissue regeneration after MI are a potential future application. Currently, the presented studies used single stimulus-responsive materials for 4D printing. Temperature and light are the most commonly used stimuli in the fabrication of 4D printed cardiovascular implants. Before becoming relevant in clinical use the main challenges are to address different technical issues of the underlying 3D printing process and the further development of smart materials sensitive to multiple stimuli. In extrusion-based printing approaches, enhancement of the speed and resolution, which is lower than in the photo-based printing methods, is an essential future direction. In addition, light-based printing technologies could be further improved for the use of multi-materials. Multi-material 4D printed implants would have enhanced mechanical properties and can be cost effective. However, in this approach, the different mechanical behavior of multi-material layers under tension or compression remains challenging (Rafiee et al., 2020).

Biodegradable 4D printed LAAOs exhibited a promising perspective compared to the commercial nitinol LAAOs. Further work could include the development of biodegradable smart materials as a future perspective to avoid long-term complications and the need for explantation surgeries.

The applied stimuli are an important part of 4D printing which need to be considered in the future. Current strategies using single stimulus-responsive materials for 4D printing have limitations, especially the use of direct thermal stimulation which is challenging to perform *in vivo.* One could think of replacing this with remote controlled heating systems such as NIR light or the application of a magnetic field. Furthermore, design and development of inks with a transition temperature close to body temperature will be beneficial to activate the shape transformation at body temperature without the need for external thermal stimulation.

In addition, temperature and light involving 4D processes face limitations such as low penetration depth of the light and risk of heating of the surrounding tissues, respectively. Internal stimuli, such as pH and enzymes also have their limitations. Depending on the application, 4D printed cardiovascular implants are not sensitive enough to these microenvironmental stimuli due to the formation of a protein corona layer around the implant immediately after implantation, leading to hypofunction of the system (Tapeinos et al., 2022). Therefore, the development of dual or multi-stimuli-responsive implants is required to improve the *in vivo* functionality of these 4D printed prostheses. Fabrication of bilayer or multi-layer implants, in which each layer is responsive to different stimuli, can be a solution to overcome the current shortcomings (Chu et al., 2020; Ren et al., 2021).

By the combination of self-healing and shape memory properties, it is possible to replace the traditional surgical sewing process with self-closure mechanisms in the future to prevent the need for further repair operations.

By further addressing these issues, 4D printing will become a promising approach for the next generation of smart cardiovascular implants addressing individual requirements and mimicking *in vivo* tissue dynamics.

## References

[B1] Agarwal T. Fortunato G. M. Hann S. Y. Ayan B. Vajanthri K. Y. Presutti D. (2021a). Recent Advances in Bioprinting Technologies for Engineering Cardiac Tissue. Mater. Sci. Eng. C 124, 112057. 10.1016/j.msec.2021.112057 PMC9381603 33947551

[B2] Agarwal T. Hann S. Y. Chiesa I. Cui H. Celikkin N. Micalizzi S. (2021b). 4D Printing in Biomedical Applications: Emerging Trends and Technologies. perspectives 32, 34. 10.1039/d1tb01335a 34586145

[B3] Amukarimi S. Mozafari M. (2021). 4D Bioprinting of Tissues and Organs. Bioprinting 23, e00161. 10.1016/j.bprint.2021.e00161

[B4] Ashammakhi N. Ahadian S. Zengjie F. Suthiwanich K. Lorestani F. Orive G. (2018). Advances and Future Perspectives in 4D Bioprinting. Biotechnol. J. 13 (12), 1800148. 10.1002/biot.201800148 PMC6433173 30221837

[B5] Asulin M. Michael I. Shapira A. Dvir T. (2021). One‐Step 3D Printing of Heart Patches with Built‐In Electronics for Performance Regulation. Adv. Sci. 8 (9), 2004205. 10.1002/advs.202004205 PMC8097332 33977062

[B6] Bedell M. L. Navara A. M. Du Y. Zhang S. Mikos A. G. (2020). Polymeric Systems for Bioprinting. Chem. Rev. 120 (19), 10744–10792. 10.1021/acs.chemrev.9b00834 32469510

[B7] Cabrera M. S. Sanders B. Goor O. J. G. M. Driessen-Mol A. Oomens C. W. J. Baaijens F. P. T. (2017). Computationally Designed 3D Printed Self-Expandable Polymer Stents with Biodegradation Capacity for Minimally Invasive Heart Valve Implantation: A Proof-Of-Concept Study. 3D Print. Addit. Manuf. 4 (1), 19–29. 10.1089/3dp.2016.0052 32953940 PMC7500013

[B8] Chu H. Yang W. Sun L. Cai S. Yang R. Liang W. Yu H. Liu L. (2020). 4D Printing: a Review on Recent Progresses. Micromachines 11 (9), 796. 10.3390/mi11090796 PMC7570144 32842588

[B9] Cui H. Liu C. Esworthy T. Huang Y. Yu Z. X. Zhou X. (2020). 4D Physiologically Adaptable Cardiac Patch: A 4-month *In Vivo* Study for the Treatment of Myocardial Infarction. Sci. Adv. 6 (26), eabb5067. 10.1126/sciadv.abb5067 32637623 PMC7314523

[B10] Derakhshanfar S. Mbeleck R. Xu K. Zhang X. Zhong W. Xing M. (2018). 3D Bioprinting for Biomedical Devices and Tissue Engineering: A Review of Recent Trends and Advances. Bioact. Mater. 3 (2), 144–156. 10.1016/j.bioactmat.2017.11.008 29744452 PMC5935777

[B11] Farzin A. Miri A. K. Sharifi F. Faramarzi N. Jaberi A. Mostafavi A. (2018). 3D-Printed Sugar-Based Stents Facilitating Vascular Anastomosis. Adv. Healthc. Mat. 7 (24), 1800702. 10.1002/adhm.201800702 PMC6394876 30375196

[B12] Gao B. Yang Q. Zhao X. Jin G. Ma Y. Xu F. (2016). 4D Bioprinting for Biomedical Applications. Trends Biotechnol. 34 (9), 746–756. 10.1016/j.tibtech.2016.03.004 27056447

[B13] Guvendiren M. Burdick J. A. (2012). Stiffening Hydrogels to Probe Short- and Long-Term Cellular Responses to Dynamic Mechanics. Nat. Commun. 3 (1), 792–799. 10.1038/ncomms1792 22531177

[B14] Imrie P. Jin J. (2022). Polymer 4D Printing: Advanced Shape‐change and beyond. J. Polym. Sci. 60 (2), 149–174. 10.1002/pol.20210718

[B15] Jia H. Gu S.-Y. Chang K. (2018). 3D Printed Self-Expandable Vascular Stents from Biodegradable Shape Memory Polymer. Adv. Polym. Technol. 37 (8), 3222–3228. 10.1002/adv.22091

[B16] Kabirian F. Brouki Milan P. Zamanian A. Heying R. Mozafari M. (2020). Additively Manufactured Small‐diameter Vascular Grafts with Improved Tissue Healing Using a Novel SNAP Impregnation Method. J. Biomed. Mater Res. 108 (4), 1322–1331. 10.1002/jbm.b.34481 31469517

[B17] Kabirian F. Brouki Milan P. Zamanian A. Heying R. Mozafari M. (2019). Nitric Oxide-Releasing Vascular Grafts: A Therapeutic Strategy to Promote Angiogenic Activity and Endothelium Regeneration. Acta biomater. 92, 82–91. 10.1016/j.actbio.2019.05.002 31059835

[B18] Kabirian F. Mozafari M. (2020). Decellularized ECM-Derived Bioinks: Prospects for the Future. Methods 171, 108–118. 10.1016/j.ymeth.2019.04.019 31051254

[B19] Kanu N. J. Gupta E. Vates U. K. Singh G. K. (2019). An Insight into Biomimetic 4D Printing. RSC Adv. 9 (65), 38209–38226. 10.1039/c9ra07342f 35541793 PMC9075844

[B20] Keriquel V. Oliveira H. Rémy M. Ziane S. Delmond S. Rousseau B. (2017). *In Situ* printing of Mesenchymal Stromal Cells, by Laser-Assisted Bioprinting, for *In Vivo* Bone Regeneration Applications. Sci. Rep. 7 (1), 1778–1810. 10.1038/s41598-017-01914-x 28496103 PMC5431768

[B21] Kirillova A. Maxson R. Stoychev G. Gomillion C. T. Ionov L. (2017). 4D Biofabrication Using Shape‐Morphing Hydrogels. Adv. Mat. 29 (46), 1703443. 10.1002/adma.201703443 29024044

[B22] Kloxin A. M. Kasko A. M. Salinas C. N. Anseth K. S. (2009). Photodegradable Hydrogels for Dynamic Tuning of Physical and Chemical Properties. Science 324 (5923), 59–63. 10.1126/science.1169494 19342581 PMC2756032

[B23] Kobayashi H. Hirukawa A. Tomitaka A. Yamada T. Jeun M. Bae S. (2010). Self-heating Property under Ac Magnetic Field and its Evaluation by Ac/dc Hysteresis Loops of NiFe2O4 Nanoparticles. J. Appl. Phys. 107 (9), 09B322. 10.1063/1.3355936

[B24] Kuang X. Chen K. Dunn C. K. Wu J. Li V. C. F. Qi H. J. (2018). 3D Printing of Highly Stretchable, Shape-Memory, and Self-Healing Elastomer toward Novel 4D Printing. ACS Appl. Mat. Interfaces 10 (8), 7381–7388. 10.1021/acsami.7b18265 29400445

[B25] Kuang X. Roach D. J. Wu J. Hamel C. M. Ding Z. Wang T. (2019). Advances in 4D Printing: Materials and Applications. Adv. Funct. Mat. 29 (2), 1805290. 10.1002/adfm.201805290

[B26] Kuribayashi-Shigetomi K. Onoe H. Takeuchi S. (2012). Cell Origami: Self-Folding of Three-Dimensional Cell-Laden Microstructures Driven by Cell Traction Force. PloS one 7 (12), e51085. 10.1371/journal.pone.0051085 23251426 PMC3521028

[B27] Li X. Liu B. Pei B. Chen J. Zhou D. Peng J. (2020). Inkjet Bioprinting of Biomaterials. Chem. Rev. 120 (19), 10793–10833. 10.1021/acs.chemrev.0c00008 32902959

[B28] Lin C. Liu L. Liu Y. Leng J. (2021). 4D Printing of Bioinspired Absorbable Left Atrial Appendage Occluders: a Proof-Of-Concept Study. ACS Appl. Mat. Interfaces 13 (11), 12668–12678. 10.1021/acsami.0c17192 33397086

[B29] Liu Y. Li Y. Yang G. Zheng X. Zhou S. (2015). Multi-stimulus-responsive Shape-Memory Polymer Nanocomposite Network Cross-Linked by Cellulose Nanocrystals. ACS Appl. Mat. Interfaces 7 (7), 4118–4126. 10.1021/am5081056 25647407

[B30] Lui Y. S. Sow W. T. Tan L. P. Wu Y. Lai Y. Li H. (2019). 4D Printing and Stimuli-Responsive Materials in Biomedical Aspects. Acta biomater. 92, 19–36. 10.1016/j.actbio.2019.05.005 31071476

[B31] Mei X. Zhu D. Li J. Huang K. Hu S. Li Z. (2021). A Fluid-Powered Refillable Origami Heart Pouch for Minimally Invasive Delivery of Cell Therapies in Rats and Pigs. Med 2 (11), 1253–1268. 10.1016/j.medj.2021.10.001 34825239 PMC8612456

[B32] Miao S. Cui H. Nowicki M. Lee S.-j. Almeida J. Zhou X. (2018). Photolithographic-stereolithographic-tandem Fabrication of 4D Smart Scaffolds for Improved Stem Cell Cardiomyogenic Differentiation. Biofabrication 10 (3), 035007. 10.1088/1758-5090/aabe0b 29651999 PMC5978741

[B33] Morouço P. Azimi B. Milazzo M. Mokhtari F. Fernandes C. Reis D. (2020). Four-Dimensional (Bio-) Printing: A Review on Stimuli-Responsive Mechanisms and Their Biomedical Suitability. Appl. Sci. 10 (24), 9143. 10.3390/app10249143

[B34] Murphy S. V. Atala A. (2014). 3D Bioprinting of Tissues and Organs. Nat. Biotechnol. 32 (8), 773–785. 10.1038/nbt.2958 25093879

[B35] Nagarajan B. Hu Z. Song X. Zhai W. Wei J. (2019). Development of Micro Selective Laser Melting: The State of the Art and Future Perspectives. Engineering 5 (4), 702–720. 10.1016/j.eng.2019.07.002

[B36] Nakamura K. Ueda K. Tomitaka A. Yamada T. Takemura Y. (2012). Self-heating Temperature and AC Hysteresis of Magnetic Iron Oxide Nanoparticles and Their Dependence on Secondary Particle Size. IEEE Trans. Magnetics 49 (1), 240–243. 10.1109/TMAG.2012.2226567

[B37] Patade S. R. Andhare D. D. Somvanshi S. B. Jadhav S. A. Khedkar M. V. Jadhav K. M. (2020). Self-heating Evaluation of Superparamagnetic MnFe2O4 Nanoparticles for Magnetic Fluid Hyperthermia Application towards Cancer Treatment. Ceram. Int. 46 (16), 25576–25583. 10.1016/j.ceramint.2020.07.029

[B38] Patil D. Song G. (2017). A Review of Shape Memory Material's Applications in the Offshore Oil and Gas Industry. Smart Mat. Struct. 26 (9), 093002. 10.1088/1361-665x/aa7706

[B39] Pedrotty D. M. Kuzmenko V. Karabulut E. Sugrue A. M. Livia C. Vaidya V. R. (2019). Three-dimensional Printed Biopatches with Conductive Ink Facilitate Cardiac Conduction when Applied to Disrupted Myocardium. Circulation Arrhythmia Electrophysiol. 12 (3), e006920. 10.1161/circep.118.006920 30845835

[B40] Pei E. Loh G. H. (2018). Technological Considerations for 4D Printing: an Overview. Prog. Addit. Manuf. 3 (1), 95–107. 10.1007/s40964-018-0047-1

[B41] Quanjin M. Rejab M. R. M. Idris M. S. Kumar N. M. Abdullah M. H. Reddy G. R. (2020). Recent 3D and 4D Intelligent Printing Technologies: A Comparative Review and Future Perspective. Procedia Comput. Sci. 167, 1210–1219. 10.1016/j.procs.2020.03.434

[B42] Rafiee M. Farahani R. D. Therriault D. (2020). Multi‐Material 3D and 4D Printing: A Survey. Adv. Sci. 7 (12), 1902307. 10.1002/advs.201902307 PMC7312457 32596102

[B43] Ren L. Li B. Liu Q. Ren L. Song Z. Zhou X. (2021). 4D Printing Dual Stimuli-Responsive Bilayer Structure toward Multiple Shape-Shifting. Front. Mater. 8, 134. 10.3389/fmats.2021.655160

[B44] Saidy N. T. Shabab T. Bas O. Rojas-González D. M. Menne M. Henry T. (2020). Melt Electrowriting of Complex 3D Anatomically Relevant Scaffolds. Front. Bioeng. Biotechnol. 8, 793. 10.3389/fbioe.2020.00793 32850700 PMC7396698

[B45] Serpooshan V. Hu J. B. Chirikian O. Hu D. A. Mahmoudi M. Wu S. M. (2018). “4D Printing of Actuating Cardiac Tissue,” in 3D Printing Applications in Cardiovascular Medicine (United States: Academic Press), 153–162. 10.1016/b978-0-12-803917-5.00008-0

[B46] Simińska-Stanny J. Nizioł M. Szymczyk-Ziółkowska P. Brożyna M. Junka A. Shavandi A. (2022). 4D Printing of Patterned Multimaterial Magnetic Hydrogel Actuators. Addit. Manuf. 49, 102506. 10.1016/j.addma.2021.102506

[B47] Tapeinos C. Gao H. Bauleth‐Ramos T. Santos H. A. (2022). Progress in Stimuli‐Responsive Biomaterials for Treating Cardiovascular and Cerebrovascular Diseases. Small 20, e2200291. 10.1002/smll.202200291 35306751

[B48] Ter Schiphorst J. Van Den Broek M. De Koning T. Murphy J. N. Schenning A. P. H. J. Esteves A. C. C. (2016). Dual Light and Temperature Responsive Cotton Fabric Functionalized with a Surface-Grafted Spiropyran-NIPAAm-Hydrogel. J. Mat. Chem. A 4 (22), 8676–8681. 10.1039/c6ta00161k

[B49] Thomas T. Rubfiaro A. S. Nautiyal P. Brooks R. Dickerson D. He J. (2020). Extrusion 3D Printing of Porous Silicone Architectures for Engineering Human Cardiomyocyte-Infused Patches Mimicking Adult Heart Stiffness. ACS Appl. Bio Mat. 3 (9), 5865–5871. 10.1021/acsabm.0c00572 35021814

[B50] Tomov M. L. Perez L. Ning L. Chen H. Jing B. Mingee A. (2021). A 3D Bioprinted *In Vitro* Model of Pulmonary Artery Atresia to Evaluate Endothelial Cell Response to Microenvironment. Adv. Healthc. Mater. 10 (20), 2100968. 10.1002/adhm.202100968 PMC8823098 34369107

[B51] Touri M. Kabirian F. Saadati M. Ramakrishna S. Mozafari M. (2019). Additive Manufacturing of Biomaterials − the Evolution of Rapid Prototyping. Adv. Eng. Mat. 21 (2), 1800511. 10.1002/adem.201800511

[B52] Van Oosten C. L. Bastiaansen C. W. M. Broer D. J. (2009). Printed Artificial Cilia from Liquid-Crystal Network Actuators Modularly Driven by Light. Nat. Mater 8 (8), 677–682. 10.1038/nmat2487 19561599

[B53] Wallin T. J. Pikul J. Shepherd R. F. (2018). 3D Printing of Soft Robotic Systems. Nat. Rev. Mater 3 (6), 84–100. 10.1038/s41578-018-0002-2

[B54] Wan X. Wei H. Zhang F. Liu Y. Leng J. (2019). 3D Printing of Shape Memory Poly( D , L ‐lactide‐ Co ‐trimethylene Carbonate) by Direct Ink Writing for Shape‐changing Structures. J. Appl. Polym. Sci. 136 (44), 48177. 10.1002/app.48177

[B55] Wang C. Yue H. Liu J. Zhao Q. He Z. Li K. (2020). Advanced Reconfigurable Scaffolds Fabricated by 4D Printing for Treating Critical-Size Bone Defects of Irregular Shapes. Biofabrication 12 (4), 045025. 10.1088/1758-5090/abab5b 32736373

[B56] Wang Y. Cui H. Esworthy T. Mei D. Wang Y. Zhang L. G. (2021a). Emerging 4D Printing Strategies for Next‐generation Tissue Regeneration and Medical Devices. Adv. Mater. 10.1002/adma.202109198 34951494

[B57] Wang Y. Cui H. Wang Y. Xu C. Esworthy T. J. Hann S. Y. (2021b). 4D Printed Cardiac Construct with Aligned Myofibers and Adjustable Curvature for Myocardial Regeneration. ACS Appl. Mat. Interfaces 13 (11), 12746–12758. 10.1021/acsami.0c17610 PMC9554838 33405502

[B58] Wei H. Zhang Q. Yao Y. Liu L. Liu Y. Leng J. (2017). Direct-write Fabrication of 4D Active Shape-Changing Structures Based on a Shape Memory Polymer and its Nanocomposite. ACS Appl. Mat. Interfaces 9 (1), 876–883. 10.1021/acsami.6b12824 27997104

[B59] Wei M. Gao Y. Li X. Serpe M. J. (2017). Stimuli-responsive Polymers and Their Applications. Polym. Chem. 8 (1), 127–143. 10.1039/c6py01585a

[B60] Willemen N. G. Morsink M. A. Veerman D. da Silva C. F. Cardoso J. C. Souto E. B. (2022). From Oral Formulations to Drug-Eluting Implants: Using 3D and 4D Printing to Develop Drug Delivery Systems and Personalized Medicine. Bio-Design Manuf., 5, 85–106. 10.1007/s42242-021-00157-0

[B61] Wu Y. Vida V. L. Zheng M. Yang J. (2021). “Progress and Prospects of Cardiovascular 3D Printing,” in Cardiovascular 3D Printing (Singapore: Springer), 179–185. 10.1007/978-981-15-6957-9_13

[B62] Wu Z. Zhao J. Wu W. Wang P. Wang B. Li G. (2018). Radial Compressive Property and the Proof-Of-Concept Study for Realizing Self-Expansion of 3D Printing Polylactic Acid Vascular Stents with Negative Poisson's Ratio Structure. Materials 11 (8), 1357. 10.3390/ma11081357 PMC6119892 30082593

[B63] Zhang C. Cai D. Liao P. Su J.-W. Deng H. Vardhanabhuti B. (2021). 4D Printing of Shape-Memory Polymeric Scaffolds for Adaptive Biomedical Implantation. Acta Biomater. 122, 101–110. 10.1016/j.actbio.2020.12.042 33359298 PMC7897283

[B64] Zhou Y. Zhou D. Cao P. Zhang X. Wang Q. Wang T. (2021). 4D Printing of Shape Memory Vascular Stent Based on βCD‐g‐Polycaprolactone. Macromol. Rapid Commun. 42, e2100176. 10.1002/marc.202100176 34121258

